# Effects of violence on executive function: a neuropsychological and predictive analysis in victims of the armed conflict in Colombia

**DOI:** 10.3389/fnhum.2025.1737110

**Published:** 2026-01-21

**Authors:** Loida Camargo, Aida Patricia Manjarrés, Marina B. Martínez-Gonzalez

**Affiliations:** 1 Faculty of Human and Social Sciences, Universidad de la Costa, Barranquilla, Colombia; 2 Faculty of Medicine, Universidad de Cartagena, Grupo Neurociencia y Salud Global, Cartagena, Colombia

**Keywords:** armed conflict, cognitive flexibility, confirmatory factor analysis (CFA), executive funcion, inhibitory control, MIMIC models, trauma

## Abstract

**Introduction:**

Exposure to violence has been associated with alterations in how the brain organizes thought, regulates emotions, and guides behavior. In Colombia, decades of armed conflict have generated heterogeneous patterns of cognitive vulnerability, yet evidence on executive and emotional functioning in adult victims remains limited.

**Methods:**

This cross-sectional study compared executive and emotional functioning between victims and non-victims of the Colombian armed conflict. A total of 300 middle-aged adults from the Department of Bolívar were assessed using a neuropsychological battery (MMSE, Tower of Hanoi, Wisconsin Card Sorting Test, Stroop Test, and Frontal Assessment Battery) and emotional screening instruments (GAD-7 and PHQ-2). Executive function was modeled as a latent construct using confirmatory factor analysis and Multiple Indicators Multiple Causes (MIMIC) models adjusted for sociodemographic covariates. Random Forest was implemented as a complementary, assumption-free analytic strategy.

**Results:**

Victims showed significantly poorer performance in planning, cognitive flexibility, and inhibitory control (β = –0.178; *p* = 0.024). Educational attainment emerged as the strongest predictor of executive functioning (β = –0.249; *p* < 0.001). The factorial model showed modest fit (CFI = 0.76), reflecting ecological and educational heterogeneity. Machine learning analyses converged with latent-variable results, identifying exposure to violence and structural inequalities as the strongest predictors of executive variability, whereas emotional factors played a marginal role.

**Discussion:**

The findings delineate a cognitive profile associated with conflict-related victimization, characterized by vulnerabilities in executive control processes. These results underscore the need for clinical and public policy interventions that integrate neuroscientific approaches to strengthen planning and decision-making capacities in populations affected by armed conflict.

## 1 Introduction

Armed conflict has been associated with enduring neuropsychological consequences that extend beyond the visible wounds of war (Weisleder and Rublee, 2018). Exposure to violence has been linked to alterations in neural circuits associated with executive control, emotional regulation, and decision-making (Zucchelli and Ugazio, 2019). In contexts like Colombia, where decades of internal conflict have deeply marked civilian populations, understanding these cognitive sequelae is crucial for designing effective rehabilitation strategies.

Executive functions are not a single skill but a family of interrelated control processes that support goal-directed behavior. Contemporary models converge on the idea of a limited set of core functions—inhibition, cognitive flexibility and updating or working memory—that share a common control mechanism while retaining partially dissociable components (Friedman and Miyake, 2017; Diamond, 2020). From a clinical perspective, these functions materialize in the capacity to formulate goals, plan action sequences, monitor performance and flexibly adapt to feedback (Jones and Graff-Radford, 2021). Our initial test battery was therefore selected to sample these domains: the Stroop Test to capture inhibitory control and resistance to interference; the Wisconsin Card Sorting Test to evaluate cognitive flexibility and perseveration; the Tower of Hanoi to assess planning, sequencing and strategic monitoring; and the Frontal Assessment Battery to probe inhibitory control, abstraction, motor programming and environmental autonomy. Together, these tasks were intended to approximate the multifaceted executive system described in the major cognitive and neuropsychological models.

Chronic exposure to violence and psychosocial stress has been consistently linked to alterations in these prefrontal systems (Arnsten et al., 2015). Experimental and epidemiological studies suggest that sustained threat, instability and resource deprivation can reshape fronto-limbic networks, weaken top–down control and favor rigid, perseverative patterns of responding (Danese et al., 2017; Nicholson and Lutz, 2017). In this neurobiological framework, trauma and structural inequality do not only increase the risk of emotional disorders; they may also erode the executive “architecture” that underpins planning, decision-making and adaptive regulation in daily life. However, evidence from Latin American post-conflict settings remains scarce, and very few studies have attempted to examine these processes using latent-variable models and modern analytic tools.

Our study was conceived as an exploratory contribution to this gap. Because our primary aim was to evaluate executive functioning in this population, we applied an established theoretical model rather than attempting to propose or validate a new construct. We therefore used a small set of classic prefrontal-sensitive measures (Frontal Assessment Battery, Wisconsin Card Sorting Test, Stroop Test, and Tower of Hanoi) to examine whether any meaningful approximation of the theoretical structure of executive control could emerge under real-world conditions of educational heterogeneity and limited literacy. In this context, the confirmatory factor and MIMIC models served as tools to summarize shared variance across tasks and to generate preliminary hypotheses about how structural disadvantage and conflict exposure may be associated with executive profiles. Given the ecological complexity and educational heterogeneity reflected in the factorial model (CFI = 0.76), we complemented these analyses with machine-learning techniques—such as Random Forest—that do not require assumptions of normality, linearity, or strict construct coherence, thereby allowing a more flexible exploration of predictive patterns in contexts of structural vulnerability.

## 2 Materials and methods

### 2.1 Design

This study employed a cross-sectional comparative design conducted in the Department of Bolívar, Colombia. Its objective was to examine variations in executive function and emotional symptoms among communities that had experienced different trajectories of violence and social vulnerability.

### 2.2 Sample size and participants

A community-wide recruitment strategy was implemented in the rural district of San Agustín (San Juan de Nepomuceno). All adult residents of the community were invited to participate, and the research team conducted two full-day medical and psychological assessment brigades. Although 250 adults expressed interest, only 150 attended and completed the assessments, largely due to the long travel distances within the corregimiento and work-related constraints. These 150 participants therefore represent the entirety of the accessible adult population who were able to attend the scheduled evaluations, rather than a selective subsample.

Victim status was operationalized through structured self-report. During the sociodemographic interview, participants indicated whether they had experienced displacement, threats, armed confrontation, or other legally recognized conflict-related events. Self-report has been widely used as a valid approach for identifying victims of political violence in community studies, particularly in contexts where official records are incomplete or underreported (Wilker et al., 2015).

To create a comparison group with similar structural vulnerability, a 1:1 matched recruitment was conducted in two peripheral and socioeconomically deprived neighborhoods of Cartagena (Henequén and Isla de León). Both settings correspond to stratum 1, characterized by poverty and high social fragility. Although geographic contexts differed, the sampling intentionally sought to approximate theoretical comparability by selecting urban areas with socioeconomic deprivation comparable to the rural community.

The resulting sample of 300 adults reflects both the full accessible population in the rural setting and a matched vulnerable urban cohort. Given the exploratory nature of this study and the logistical challenges inherent to fieldwork in post-conflict areas, the sample size is adequate for preliminary latent-variable exploration, while its limitations for confirmatory structural modeling are acknowledged explicitly in later sections.

### 2.3 Geographic and logistical setting

*San Juan de Nepomuceno* is located approximately 1 h by car from *Cartagena de Indias*, both within the Department of Bolívar. However, the *San Agustín* district lacks a direct road connection to the municipal center. Reaching the community requires a 4-h overland journey from Cartagena to *Tenerife* (passing through San Juan de Nepomuceno, in the neighboring Department of Magdalena), followed by a 30-min river crossing by boat.

Due to persistent security risks and the complex geography of the area, the research required logistical and safety coordination with the Colombian Public Forces. This collaboration ensured the research team’s safe access to the community and the participants’ protection during fieldwork activities.

### 2.4 Instruments

#### 2.4.1 A comprehensive set of instruments was used for data collection:

*Mini-mental state examination (MMSE):* Used for general cognitive screening. It is recommended by the Colombian Ministry of Health for first-level cognitive assessment (Ministerio de Salud y Protección Social, 2018). The MMSE evaluates attention, calculation, language, visuospatial skills, and short-term and delayed memory. The instrument has been validated and widely used in Colombia, demonstrating appropriate psychometric properties in adults and older populations (Rosselli et al., 2000).

*Stroop test (Stroop, 1935): Measures selective attention, inhibition, and cognitive flexibility by examining an individual’s ability to suppress automatic responses to conflicting color–word stimuli. Its clinical utility and sensitivity to executive impairments have been confirmed in Colombian populations (Rodríguez-Barreto et al., 2016; Bajaj et al., 2013; Coelli et al., 2016; Scarpina and Tagini, 2017).*

*Tower of Hanoi (Lucas, 1883): Frequently used in adult neuropsychological assessment as a measure of planning and strategic problem solving. We administered the three-disk version, which offers a standardized evaluation of sequencing and monitoring processes and has been shown to capture core planning components in adult samples (Welsh et al., 1999; Wood and Worthington, 2017).*

Wisconsin card sorting test—48-card version:

*Frontal assessment battery*: Developed by Dubois et al. (2000), this brief tool assesses various executive components associated with the prefrontal cortex, including inhibition, environmental autonomy, motor programming, verbal fluency, imitation behavior, and abstract reasoning. In Colombian samples, it has shown satisfactory discriminant validity between patients with and without frontal damage and has been included in several clinical studies (Ardila et al., 2010).

#### 2.4.2 Emotional measures

- *Generalized anxiety disorder scale (GAD-7)* (Spitzer et al., 2006) was used to assess anxiety-related symptoms such as worry, restlessness, irritability, difficulty relaxing, and nervousness. Its validation in Colombia confirmed adequate factorial structure and high internal consistency in both general and medical populations (Camargo et al., 2023).
- *Patient health questionnaire-2 (PHQ-2)* (Löwe et al., 2005) was used to screen for depressive symptoms including anhedonia, low mood, agitation, poor concentration, fatigue, and sleep or appetite disturbances. The PHQ-2 has been widely used in Colombian primary care settings, with satisfactory sensitivity and specificity for detecting depression (Cassiani-Miranda et al., 2021).

##### 2.4.2.1 Sociodemographic questionnaire

A custom form collected data on marital status, access to public and health services, ethnicity, residence, occupation, age, sex, and exposure to violence. The instrument was pilot-tested to ensure cultural and linguistic appropriateness for the target population.

### 2.5 Fieldwork procedures

Data collection was conducted in both municipalities in close coordination with local authorities and community leaders. The study protocol was approved by an ethics committee document number 297 of Costa University and all participants provided informed consent prior to participation.

Between January and June 2025, four field visits were carried out—three in Cartagena and one in San Juan de Nepomuceno. Each visit involved community-wide calls for participation in coordination with local leaders. During these sessions, participants received general and specialized medical care, psychological support, and the neuropsychological assessments described above.

The two Cartagena neighborhoods selected—Henequén and Isla de León—were chosen a priori because they are classified as stratum 1, with high levels of socioeconomic vulnerability, marked informal employment, and cultural constructs broadly comparable to those of the rural community, despite the expected differences associated with closer proximity to the urban center.

### 2.6 Data analysis

Executive function was modeled as a latent construct using the FAB, WCST, and the Tower of Hanoi. The Stroop Test was administered but excluded from the analyses due to literacy-related discontinuation. A Confirmatory Factor Analysis (CFA) was conducted as an exploratory examination of whether the established theoretical structure of executive control could be represented in this population; given the modest fit obtained, latent estimates were interpreted with caution. Measurement invariance (configural, metric, and scalar) across victim and non-victim groups was evaluated using ΔCFI ≤ 0.01 as criterion.

A Multiple Indicators Multiple Causes (MIMIC) model was then used to estimate the associations between victim status and the latent executive factor. Covariates included age, years of schooling, occupation (formal vs. informal), literacy, ethnicity, residence, and health-insurance status. These variables were incorporated to contextualize group differences, acknowledging that residual confounding may persist despite statistical adjustment.

For emotional symptoms, the unidimensional structure of the GAD-7 was examined and its invariance tested across groups. Depressive symptoms were screened using the PHQ-2. Machine-learning procedures (Causal Forests, Elastic Net, and Gaussian Mixture Models) were applied in an exploratory manner and tuned through internal cross-validation.

## 3 Results

Victims of the armed conflict (*n* = 150) exhibited a more fragile sociodemographic profile compared to non-victims (*n* = 150). Nearly half of the victims were men (46.7% vs. 28.0%; *p* = 0.0019), and a considerable proportion belonged to ethnic minority communities—Indigenous, Afro-descendant, or *palenquera*—(*p* = 0.0192). A clear gap emerged in healthcare access: only two out of three victims were covered by a medical insurance scheme (66%), compared with more than 85% of non-victims (*p* = 0.0002). Differences were also evident in household living conditions; most victims had access to only one or two basic public services, whereas in Cartagena, households typically had four or five (*p* < 0.0001).

Educational disparities were concentrated at the lower levels. Although middle and higher education rates were similar, non-victims more frequently reported completing primary education (*p* = 0.0003). Formal employment was low in both groups; however, victims were significantly more dependent on state subsidies (17.3% vs. 6.7%; *p* = 0.0030). Marital status and the presence of chronic diseases showed no significant differences (see Table 1).

**TABLE 1:** Sociodemographic characteristics of participants according to victimization status.

Variable	Category	Victims (*n* = 150)	Not victims (*n* = 150)	*p*-value (Chi^2^/mann–whitney U)
Age	Average (SD)	55.2 ( ± 15.6)	50.0 ( ± 16.8)	0.0045†
Gender	Male	70 (46.7%)	42 (28.0%)	0.0019
	Female	79 (52.7%)	108 (72.0%)	
Marital status	Single	37	47	0.0735
	Divorced	48	29	
	Common-law marriage	15	11	
	Other	41	48	
	Ns/Nc	9	15	
Ethnic group	Yes	13	25	0.0192
	No	131	125	
	Ns/Nc	6	0	
Specific ethnic group	Indigenous	9	15	0.0308
	Afro-descendant	10	13	
	Raizal	2	0	
	Palenquero	7	0	
	None	120	121	
	Ns/Nc	2	0	
Access to healthcare	Yes	99 (66.0%)	128 (85.3%)	0.0002
	No	51	22	
Education	Elementary school	87	52	0.0003
	Middle school	55	87	
	High school	8	8	
High school diploma or highers	No	109	117	0.449
	Yes	37	31	
Government support	Yes	26 (17.3%)	10 (6.7%)	0.0030
	No	124	140	
Formal employment	Yes	39	39	1.000
	No	111	111	
Chronic illness	Yes	38	40	0.8953
	No	112	110	
Public services	1 service	10	3	<0.0001
	2 services	114	37	
	3 services	4	15	
	4 services	10	41	
	5 services	11	54	

†Mann–Whitney U test. All other comparisons were performed using the Chi-square test.

General cognitive performance, assessed through the MMSE, was low in both groups (median = 24 vs. 25). The total score did not differ significantly, but notable differences appeared in the orientation (*p* < 0.001) and recall domains (*p* = 0.029). In contrast, the executive tests showed pronounced disparities: victims took longer to complete the Tower of Hanoi (*p* < 0.001), committed more perseverative errors on the Wisconsin Card Sorting Test (*p* < 0.001), and obtained lower FAB scores (median = 10 vs. 12; *p* < 0.001), particularly in motor sequencing, verbal fluency, and inhibitory control. The Stroop Test, although initially included, could not be analyzed because more than half of the victims discontinued the task due to reading difficulties (see Table 2).

**TABLE 2:** Comparison of cognitive and emotional functioning between victims and non-victims of armed conflict.

Domain	Variable	Median (victims)	Median (Non-victims)	*p* (Mann–Whitney U)	*r*	Interpretation
MMSE	Orientation	8.0	9.0	<0.001	–0.23	Small–moderate
	Memory	3.0	3.0	0.02	–0.08	Minimal
	Attention/calculation	4.0	4.0	0.52	–0.04	ns
	Recall	2.0	2.0	0.03	–0.14	Small
	Language	8.0	8.0	0.68	0.03	ns
	Total	24.0	25.0	0.02	–0.15	Small
Tower of Hanoi	Number of moves	11	9	< 0.001	–0.29	Small–moderate
	Errors	8	6	0.16	0.05	ns
Wisconsin	Perseverative errors	24.5	16.5	<0.001	–0.29	Small–moderate
	Random responses	7.0	0.0	0.01	0.18	Small
FAB	Verbal fluency	2.0	3.0	0.001	–0.21	Small
	Motor sequences	2.0	3.0	<0.001	–0.29	Small–moderate
	Conflicting instructions	1.0	2.0	<0.001	–0.24	Small
	Go/No-Go	1.0	2.0	<0.001	–0.22	Small
	Total	10.0	12.0	<0.001	–0.34	Moderate
GAD-7	Total anxiety	5.0	7.0	0.001	–0.21	Small–moderate
PHQ-2	Total depression	1.0	1.0	0.80	0.02	ns

MMSE, Mini-Mental State Examination; FAB, Frontal Assessment Battery; GAD-7, Generalized Anxiety Disorder-7; PHQ-2, Patient Health Questionnaire-2. Comparisons using Mann–Whitney U test. r corresponds to the effect size according to Rosenthal.

The Confirmatory Factor Analysis suggested only a limited and coarse approximation to the theoretical structure of executive control, rather than a true representation of it. The strongest loadings were observed for the Tower of Hanoi (λ = 0.752) and the reversed random-response indicator (λ = 0.854). Reliability was modest (ω = 0.42), and global fit indices (CFI = 0.76; RMSEA = 0.204) reflected both the heterogeneity of the population and the constraints associated with field-based neuropsychological assessment. Accordingly, latent estimates were interpreted with caution, and the model was used primarily as a summary of shared variance across tasks rather than as a definitive validation of the executive construct, with all parameter estimates reported in Table 3.

**TABLE 3:** Confirmatory factor model of executive function (CFA).

Indicator	Standardized loading (λ)	Explained variance (R^2^)	*p*-value
FAB—similarities	0.23	0.05	0.067
FAB—verbal fluency	0.37	0.13	<0.001
FAB—motor sequences	0.43	0.18	<0.001
FAB—conflicting instructions	0.45	0.21	<0.001
FAB—Go/No-Go	0.38	0.14	<0.001
FAB—prehension behavior	0.29	0.08	0.005
WCST—categories achieved	0.54	0.29	<0.001
Tower of Hanoi—correct moves	0.75	0.57	<0.001
Tower of Hanoi—errors	–0.59	0.35	<0.001
Tower of Hanoi—perseverations	–0.58	0.34	<0.001
Random responses (reversed)	0.85	0.73	<0.001

Overall fit indices: ω = 0.42; αα = 0.33; α_ord = 0.53; AVE = 0.28; CFI = 0.76; TLI = 0.70; RMSEA = 0.20; SRMR = 0.15. Confirmatory factor model of executive function. Standardized loadings (λ) indicate the contribution of each indicator to the latent factor. The model showed moderate overall fit (CFI = 0.76; RMSEA = 0.20; ω = 0.42).

The MIMIC model showed that victim status was associated with lower latent executive performance (β = –0.178; *p* = 0.024). Educational attainment displayed an even stronger association with the latent factor (β = –0.249; *p* < 0.001), and formal employment was linked to better executive scores (β = 0.198; *p* = 0.005). Access to medical care and psychosocial support showed small positive trends (*p* ≈ 0.07–0.09). These associations indicate that differences in executive functioning reflect a combination of conflict exposure and broader structural conditions, recognizing that statistical adjustment cannot fully disentangle their overlapping influences; detailed model estimates are provided in the MIMIC results.

Emotional findings followed a distinct pattern. In the GAD-7, victims showed slightly lower raw anxiety scores compared with non-victims. Given the lack of full scalar invariance, no latent mean comparisons were performed, and interpretations are based exclusively on observed scores and model associations. No significant differences were observed in depressive symptoms (Δ = –0.187; *p* = 0.309). The latent correlation between anxiety and depression was high (*r* = 0.773; *p* < 0.001; see Table 4).

**TABLE 4:** Confirmatory models of anxiety (GAD-7) and depression (PHQ-2).

Instrument/ítem	Standardized loading (λ)	Explained variance (R^2^)	*p*-value
**GAD-7 (anxiety)**			
Nervousness	0.70	0.49	<0.001
Uncontrollable worry	0.74	0.55	<0.001
Excessive worry	0.85	0.72	<0.001
Difficulty relaxing	0.73	0.53	<0.001
Restlessness	0.76	0.58	<0.001
Irritability	0.49	0.24	<0.001
Anticipatory fear	0.69	0.48	<0.001
Composite reliability (ω)	0.87		
Global explained variance (R^2^)	0.72		
**PHQ-2 (depression)**			
Depressed mood	0.70	0.49	<0.001
Anhedonia	0.89	0.79	<0.001
Composite reliability (ω)	0.69		

Latent correlation between factors: *r* = 0.773; *p* < 0.001. Confirmatory models of anxiety (GAD-7) and depression (PHQ-2). The loadings (λ) reflect the strength of association of each item with its respective construct. A significant correlation was observed between anxiety and depression (*r* = 0.773; *p* < 0.001).

Exploratory machine-learning analyses were conducted to examine whether data-driven models would converge with the patterns observed in the CFA and MIMIC results. These algorithms were not used for causal inference, but rather to assess the relative contribution of sociodemographic, contextual, and emotional variables to the prediction of executive scores under different modeling assumptions. Across models, sociodemographic and violence-related variables explained most of the variance in executive performance, whereas health and emotional measures contributed minimally. Among the techniques applied, the Random Forest achieved the lowest prediction error, suggesting that in this adult sample, variability in executive functioning was most consistently associated with social context and conflict-related conditions.

## 4 Discussion

Our objective was to examine differences in executive and emotional processing between two adult groups from the same regional context—individuals who were victims and non-victims of the Colombian armed conflict. Although both groups shared low socioeconomic conditions, disparities in access to healthcare, public services, and state support revealed that structural inequality remains a central determinant of vulnerability. These contextual factors frame cognitive performance and help explain part of the variability observed between groups. However, the associations identified suggest that structural adversity alone may not fully account for the executive profile observed in victims and that conflict-related experiences may intersect with these broader social disadvantages (Organisation for Economic Co-operation and Development [OECD], 2022; Fernández-Ortiz and Peñaloza-Quintero, 2025).

Global performance on the MMSE did not differ between groups, suggesting that general cognition remained relatively preserved. Yet this apparent similarity likely reflects the instrument’s limitations. The MMSE is not sensitive to executive dysfunction—it privileges memory and orientation and often fails to detect deficits in planning or inhibitory control (Hoshi et al., 2022). Therefore, the absence of global differences does not contradict subsequent findings: impairments emerge precisely when the underlying processes of decision-making are examined (Davis and Allen, 2013; Spencer et al., 2013; Devenney and Hodges, 2017).

The high proportion of missing data in the Stroop test was not a technical issue but an educational marker. Functional literacy was limited in many participants, even among those who had completed formal schooling. This finding underscores a key point: actual literacy predicts cognitive performance more accurately than years of education (de Resende et al., 2022). In contexts where educational quality is heterogeneous, “nominal schooling” does not necessarily translate into effective reading competence, which directly constrains executive functioning (Serper et al., 2014; Peng et al., 2022).

Specific tests did reveal clear differences. Victims showed reduced cognitive flexibility and greater perseveration on the Wisconsin Card Sorting Test, lower overall FAB scores, and slower performance on the Tower of Hanoi. These results suggest a pattern of difficulties in organizing, inhibiting, and adapting responses. Evidence from other populations exposed to violence points in similar directions: repeated adversity has been associated with alterations in prefrontal control mechanisms, reducing the ability to generate new strategies and adjust behavior based on feedback (Sorel et al., 2023; Nicholson and Lutz, 2017; Danese et al., 2017). In our context, however, these associations must be interpreted within the broader landscape of inequality, opportunity gaps, and accumulated disadvantage.

The confirmatory factor analysis was conducted as an exploratory approach to examine whether a pre-established theoretical model of executive control could be represented in this heterogeneous sample. The modest model fit indicates that the latent structure should be interpreted cautiously and used primarily as a summary of shared variance across tasks rather than as a definitive validation of executive function in this context. Accordingly, we treated the CFA as an exploratory summary of shared variance rather than as a strict test of construct validity. While the global fit indices reflect the sample’s educational heterogeneity and ecological limitations, we interpreted the factor structure not as a strict psychometric validation, but rather as an assessment of the construct’s theoretical transferability within this context. Crucially, we verified that the direction and magnitude of the associations in the latent model broadly converged with those observed in the raw scores, provides descriptive convergence with theory-driven models under a different set of assumptions, without implying confirmatory validation. Given the exploratory aims of the study, the factorial model was not modified by removing indicators. Our intention was not to fine-tune a measurement model or validate a new construct, but to examine how classical executive-function tasks perform in a population characterized by low literacy and substantial educational heterogeneity. In this context, the limited model fit reflects not only psychometric constraints but also the challenges of transferring instruments developed in highly educated settings. For these reasons, the CFA was used primarily as a summary of shared variance, and its results were interpreted with appropriate caution.

Importantly, the modest CFA fit should not be interpreted as a technical failure of the data, but can also be empirical indication that the executive-function construct—developed and validated mainly in highly educated Western populations—shows reduced cohesion in contexts of low literacy and heterogeneous schooling. This pattern aligns with the cognitive differentiation hypothesis, which proposes that in populations with limited educational opportunities, cognitive abilities tend to be less fractionated and more interdependent (Breit et al., 2022; Soto-Añari and Cáceres-Luna, 2012). In this sense, the weaker factorial structure observed here is compatible with the idea that, in structurally deprived settings, executive components may not separate as cleanly as the theoretical model predicts.

The MIMIC analysis showed that victimization, lower schooling, and informal employment were associated with lower latent executive scores, whereas formal employment and access to healthcare were linked to better performance, although some of these trends were marginal. These associations emphasize that executive functioning reflects an interplay of socioeconomic, educational, and contextual factors. We were aware that schooling differences could influence the measurement of executive functioning. For this reason, years of education were included as a covariate to reduce, as far as possible, the risk of confounding. Nonetheless, a degree of residual influence is likely to persist, mediated by factors not fully captured by formal schooling—such as limited cognitive stimulation, restricted learning opportunities, and cumulative exposure to risks associated with socio-economic vulnerability, including inadequate nutrition and environmental toxins (Torres et al., 2025).

Emotional symptoms showed a modest difference between groups, with victims reporting slightly lower anxiety levels in raw scores. However, because full scalar invariance was not achieved for the GAD-7, these differences should be interpreted cautiously and no conclusions can be drawn about latent (measurement-adjusted) differences No group differences were observed in depression, which may reflect resilience processes accumulated over time (Gómez-Restrepo et al., 2025; Madero et al., 2025).

The exploratory machine-learning analysis provided a complementary perspective. These models were not intended to establish causation, but to evaluate the relative contribution of different predictors under data-driven assumptions. Across supervised algorithms, sociodemographic and conflict-related variables accounted for most of the variance in executive functioning, whereas health and emotional factors contributed minimally. The Random Forest achieved the lowest mean squared error, although differences with LASSO were small. Taken together, these findings align with the broader pattern derived from theory-driven analyses: variability in executive performance appears closely associated with accumulated structural disadvantages and conflict-related conditions, acknowledging that these influences are deeply intertwined and cannot be completely disentangled statistically.

While the fit indices of the latent factor model were suboptimal—reflecting the complexity of measuring executive function in diverse, non-WEIRD populations—the internal consistency of the findings is strengthened by their convergence with the machine-learning analyses. The fact that these supervised and unsupervised algorithms identified similar patterns of deficit suggests that the observed associations are unlikely to be mere artifacts of model misspecification and may reflect a meaningful neuropsychological pattern in this population.

Across analytic approaches, schooling emerged as the variable most strongly associated with executive performance, even more than victimization status. This pattern aligns with evidence from Latin American cohorts showing that educational opportunities—not only the number of years spent in school—play a central role in shaping cognitive resilience and long-term executive trajectories (Soto-Añari and Cáceres-Luna, 2012; Branco et al., 2014). In post-conflict settings, this suggests that strengthening educational quality and adult literacy may be among the most impactful pathways to support cognitive functioning and daily autonomy.

It is crucial to interpret the observed executive deficits within the broader ecological context of the conflict. In this region, victimization is closely intertwined with structural neglect and geographic isolation. Therefore, the pattern observed appears compatible with a “cumulative burden” framework, in which the biological consequences of violence and environmental deprivation likely co-occur and jointly contribute to the observed executive profile. Crucially, despite this overlap, our unsupervised machine-learning analyses (Gaussian Mixture Models)—which operate without a priori group assumptions—also identified profiles in which victimization status clustered with lower executive performance. Given the sample size and cross-sectional design, however, these findings remain descriptive and do not strengthen causal inference. This suggests that the association between conflict exposure and executive performance remains detectable even within this complex background of structural disadvantage.

Because Random Forest models do not rely on linearity, normality, or measurement invariance, their consistency with the patterns observed in the CFA and MIMIC models offers complementary support for the associations identified under different sets of assumptions. Importantly, we interpret this convergence as descriptive rather than confirmatory; nonetheless, the fact that two methodologically distinct approaches highlight the same sociodemographic predictors reinforces the robustness of the observed associations.

From both clinical and public health perspectives, these results highlight the need to incorporate cognitive rehabilitation within victim care programs. Interventions targeting executive functions not only improve test performance but also enhance autonomy and the ability to plan daily life. Evidence shows that cognitive training programs can strengthen planning, flexibility, and inhibitory control, improving functional outcomes and emotional wellbeing (Bomyea et al., 2024). Without reinforcing this component, social and economic reintegration is likely to remain remains incomplete.

### 4.1 Limitations

The sample size was modest and may have affected the stability of the confirmatory models, particularly the CFA, which showed poor global fit and therefore limits the precision of latent estimates. Although years of schooling were included as a covariate, educational attainment does not fully capture the heterogeneity in literacy and learning opportunities across groups. Therefore, some degree of residual confounding is likely and should be considered when interpreting group differences. Administering neuropsychological tests in populations with low literacy also highlighted measurement challenges: the Stroop Test could not be completed by a substantial proportion of participants, reducing the breadth of the executive assessment and emphasizing the need to adapt instruments to diverse educational contexts.

The cross-sectional design precludes causal inference. The associations observed likely reflect the intertwined influence of conflict exposure, socioeconomic disadvantage, and limited educational opportunities, which cannot be fully disentangled analytically. Several variables—especially emotional and health-related ones—relied on self-report and may be influenced by culturally shaped response styles. We did not adjust *p*-values for multiple comparisons; therefore, findings with *p*-values close to 0.05 should be interpreted cautiously. Additionally, the exploratory machine-learning analyses should be interpreted with caution due to sample size constraints and their descriptive nature.

Finally, comparing a remote rural community with an urban low-income group introduces structural heterogeneity that cannot be fully resolved through statistical adjustment. Yet this heterogeneity reflects the reality in which conflict-affected populations live—diverse, unequal, and shaped by layers of disadvantage. Although imperfect, this design allows us to capture part of that lived complexity and to document cognitive patterns that might otherwise remain unseen. Despite these limitations, the study offers an initial insight into how these contextual factors relate to executive functioning and highlights the need for future longitudinal and community-based research.

## Data Availability

The datasets presented in this study can be found in online repositories. The names of the repository/repositories and accession number(s) can be found at: https://doi.org/10.5281/zenodo.17878390.
