# Correction: Effects of violence on executive function: a neuropsychological and predictive analysis in victims of the armed conflict in Colombia

**DOI:** 10.3389/fnhum.2026.1803425

**Published:** 2026-04-02

**Authors:** Loida Camargo, Aida Patricia Manjarrés, Marina B. Martínez-Gonzalez

**Affiliations:** 1 Faculty of Human and Social Sciences, Universidad de la Costa, Barranquilla, Colombia; 2 Faculty of Medicine, Universidad de Cartagena, Grupo Neurociencia y Salud Global, Cartagena, Colombia

**Keywords:** armed conflict, cognitive flexibility, confirmatory factor analysis (CFA), executive function, inhibitory control, MIMIC models, trauma

Author Aida Patricia Manjarrés was erroneously assigned as corresponding author.

The correct corresponding author is Loida Camargo, lcamargoc@unicartagena.edu.co.

The affiliation Faculty of Medicine, Universidad de Cartagena, Grupo Neurociencia y Salud Global, Cartagena, Colombia was omitted for author Loida Camargo. This affiliation has now been added for author Loida Camargo.

The original version of this article has been updated.

